# Procrastination, Distress and Life Satisfaction across the Age Range – A German Representative Community Study

**DOI:** 10.1371/journal.pone.0148054

**Published:** 2016-02-12

**Authors:** Manfred E. Beutel, Eva M. Klein, Stefan Aufenanger, Elmar Brähler, Michael Dreier, Kai W. Müller, Oliver Quiring, Leonard Reinecke, Gabriele Schmutzer, Birgit Stark, Klaus Wölfling

**Affiliations:** 1 Department of Psychosomatic Medicine and Psychotherapy, University Medical Center of the Johannes Gutenberg University Mainz, Mainz, Germany; 2 Institute of Education, Johannes Gutenberg University Mainz, Mainz, Germany; 3 Department of Medical Psychology and Medical Sociology, University Medical Center Leipzig, Leipzig, Germany; 4 Department of Communication, Johannes Gutenberg University Mainz, Mainz, Germany; University of Vienna, School of Psychology, AUSTRIA

## Abstract

Addressing the lack of population-based data the purpose of this representative study was to assess procrastination and its associations with distress and life satisfaction across the life span. A representative German community sample (1,350 women; 1,177 men) between the ages of 14 and 95 years was examined by the short form of the General Procrastination Scale (GPS-K; 1) and standardized scales of perceived stress, depression, anxiety, fatigue and life satisfaction. As hypothesized, procrastination was highest in the youngest cohort (14–29 years). Only in the youngest and most procrastinating cohort (aged 14 to 29 years), men procrastinated more than women. As we had further hypothesized, procrastination was consistently associated with higher stress, more depression, anxiety, fatigue and reduced satisfaction across life domains, especially regarding work and income. Associations were also found with lack of a partnership and unemployment. Findings are discussed with regard to potential developmental and cohort effects. While procrastination appears to be a pervasive indicator for maladjustment, longitudinal analyses in high-risk samples (e.g. late adolescence, unemployment) are needed to identify means and mechanisms of procrastinating.

## Introduction

Procrastination, putting off intended action, is a universal phenomenon, which may be employed for many reasons (e.g. postponing action in order to avoid stress; [1]). Therefore, Steel [2] characterized procrastination as a self-regulatory failure leading to poor performance and reduced well-being. Klingsieck [3] aptly defined procrastination as “the voluntary delay of an intended and necessary and/or (personally) important activity, despite expecting potential negative consequences that outweigh the positive consequences of the delay” (p. 26). Over the last decades a considerable amount of literature has been published on procrastination and its causes and consequences in different aspects of life [2; 4]. In his seminal meta-analysis and review, Steel [2] identified task (aversiveness, delay) and personality characteristics (particularly low self-efficacy, conscientiousness, self- control and achievement motivation as well as high impulsiveness and distractibility) as determinants of procrastination, along with a potential genetic component (based on a twin study). Gröpel & Steel [5] investigated predictors of procrastination in a large internet- based study with 9,351 participants (mean age of 35 years). Based on temporal motivation theory, the results showed that goal setting, interest enhancement and energy reduced procrastination. Lack of energy was most strongly associated with procrastination, mediating the effect of interest enhancement. Goal setting appeared to be particularly important, when interest in the task was low. Additionally, they found weak negative associations of procrastination to age (*r* = -.17) and female sex (*r* = -.08).

Several studies have explored procrastinating behavior in an academic context and in the workplace. Procrastination has been most thoroughly studied in student populations, where it has been associated with increased levels of stress, anxiety, depression and poor academic performance [2; 6], but also with putting off everyday obligations [7]. In a large internet-based study (N = 22,053) on the association of procrastination with employment status and job characteristics, Nguyen et al. [8] found that procrastination was associated with lower income, shorter duration of employment and more unemployment. Employees in jobs with lower intrinsic value (e.g. recognition) and more constraints tended to procrastinate more than employees having jobs requiring higher levels of intrinsic motivation skills.

Additionally, further studies identified extreme and persistent procrastination as a risk factor for poor physical and mental well-being (e.g. [9; 10]). Procrastination as a dysfunctional form of delay was linked to delayed medical treatments and less mental health care utilization [11; 12]. In regard to socio-demographic variables, men tended to procrastinate more than women and younger compared to older participants in a large epidemiological study (N = 16,413). Additionally, procrastinators tended to be single and less well educated [13]. The found associations between procrastination and lower psychological well-being may indicate that procrastination is also linked to reduced life satisfaction [14]. As people with the tendency to procrastinate seem to be less integrated in their social and professional lives (e.g. living more often without partner, unemployed etc.), the relationship between procrastination and loneliness as an aspect of reduced life satisfaction was also considered to be worth exploring.

However, all of these results depended on the operationalization of procrastination. Among the diverse measures available, the General Procrastination Scale (GPS) by Lay [15] has been frequently used to assess “the tendency to postpone that which is necessary to reach some goal” (p. 475). However, Klingsieck & Fries [16] were not able to reproduce the postulated one-factorial structure of the original scale in a large student sample, which they explained by heterogeneity and ambiguity of some of the items (e.g. delay of getting out of bed in the morning). Based on factor analysis, Klingsieck & Fries [16] identified nine items forming a brief, one-factorial scale covering to a broad range of procrastination behaviors. In a validation study with the short scale (GPS-K), the authors found high positive correlations to the Aitken Procrastination Inventory ([17]; *r* = .75) and high negative correlations to conscientiousness (based on the BFI-10; *r* = .-.69) as well as action orientation (*r* = .-.67). Other negative associations were found to time management (*r* = .-.48), planning (*r* = .-.34), self-efficacy ([18]; *r* = .-.26) and agreeability (BFI-10; *r* = .-.20). Men procrastinated more than women.

Considering the high prevalence of procrastination and its individual and societal consequences, it is essential to explore procrastination, socio-demographic and mental health variables in a large community sample to identify risk populations. The purpose of this study was therefore to study procrastination in a representative sample of the German population across the full age range from 14 to 95 years. We intended to determine the association of procrastination to demographic and vocational factors (age, sex, education, employment, income), and a broad range of mental health characteristics, especially perceived stress, distress (depression, anxiety, fatigue) and life satisfaction across a broad set of life domains. In line with previous research findings we hypothesized that procrastination was (1) higher in younger age [13] and (2) associated with more perceived stress and distress (depression, anxiety, fatigue), reduced quality of life and less social integration [2; 9; 14].

## Method

### Participants

The present study was based on a representative survey of the German population. Data were collected by USUMA (Unabhängiger Service für Umfragen, Methoden und Analysen; Berlin) between February and April, 2014. The sample consisted of a total of 2,527 participants (1,350 women; 1,177 men) between the ages of 14 and 95 years who were recruited at 258 sample points, representing East and West Germany; the majority (79.9%) lived in the Western states of Germany. Participants, who gave informed consent, were interrogated by face-to-face-interviews by trained interviewers in their homes and independently filled out additional questionnaires in the presence of the interviewer. No incentives were offered for study participation. The survey followed ADM (Arbeitskreis Deutscher Markt- und Sozialforschungsinstitute e.V.) sampling guidelines for generating a representative sample of the German population [19]. The sampling procedure comprised three steps: First the areas were regionally stratified (1^st^ step) for identifying sampling points, where private households were selected (2^nd^ step). In the 3rd step the individual within the selected household was determined. By applying this random-route procedure the region, the households and target persons living in the households were randomly selected. Table 1 presents the data of the last official survey of the entire German population conducted in 2011 by the Statistical Federal Office showing the comparability with the data of the present sample. After contacting the selected participants in their home, 55.1% of the initial sample (4,607 households) was interviewed. The resulting quota matched other representative population samples. 46.1% of the sample was married and 58.1% lived in a partnership. The great majority had completed high school or 10^th^ grade of education (59%); 2.7% attended school. The full or part-time employment rate was 51.4% and the unemployment rate 6.0%; 28.3% received pension.

**Table 1:** Distribution of socio-demographic characteristics in the German population and in the present sample.

	German population^1)^ 2011*N* = 80 219 695	Present sample 2014 *N* = 2527
	*%*	*%*
**age groups**		
≤ 29 yrs	29.8	16.7
30–49 yrs	28.2	31.9
50–64 yrs	20.8	28.8
≥ 65 yrs	21.2	22.5
**sex**		
female	51.3	53.0
**education**		
without graduation	4.7	3.3
current in school	4.4	2.7
<10th grade	35.6	35.2
completed 10th grade	26.9	27.9
high school	28.3	30.8
**Employment**		
Employed	50.2	54.0
Unemployed	2.7	6.0
non-working	47.1	40.0

Note:

^1)^ Zensus 2011; Statistische Ämter des Bundes und der Länder (https://www.zensus2011.de/EN/Home/home_node.html)

### Ethics statement

The study and procedure, including the consent procedure, were approved by the institutional ethics review board of the University of Leipzig (Az 063-14-10032014). The ethics committee of the University of Leipzig approved the consent procedure for the whole sample including participants between 14 and 18 years. Furthermore, the study adhered to ICH-GCP-guidelines (ICH = International Conference on Harmonisation of Technical Requirements for Registration of Pharmaceuticals for Human Use; GCP = Good Clinical Practice) as well as to the guidelines of the ICC/ESOMAR (ICC = International Chamber of Commerce; ESOMAR = European Society for Opinion and Market Research) International Code of Marketing and Social Research Practice. All participants were informed of the study procedures, data collection and anonymization of all personal data. Moreover, a detailed data privacy statement was delivered by the study assistant. The present study posed a low risk to the participants, as procedures such as medical treatments, invasive diagnostics or procedures causing psychological, spiritual or social harm were not included in the present study. According to the German law, all participants provided verbal informed consent, which was noted by the trained interviewer before starting with the survey. An additional informed consent of a parent was thus not required for participants aged 14 or older.

### Measures

Demographic data included age (≥14 years), sex, marital and employment status, education and total income of household.

The German short form (GPS-K; [16]) is a one-dimensional short-form of the General Procrastination Scale (GSS) with nine items [15]. Participants rated how characteristic they consider each behaviour (e.g. “I delay the completion of certain things”) on a 4-point scale (1 = “very uncharacteristic” to 4 = “very characteristic”). As described above, it was validated in a student sample (*N* = 218). The internal consistency in the present study was Cronbach alpha = .92.

Distress, stress and fatigue were measured by the Patient Health Questionnaire (PHQ-4), the Perceived Stress Scale (PSS-10) and the Copenhagen Personal Burnout Inventory (CBI). The PHQ-4 [20] consists of two items reliably assessing the core symptoms of depressed mood and loss of interest (PHQ-2) plus two screening items of the short form of the GAD-7 (Generalized Anxiety Disorder [GAD]-2 Scale): “Feeling nervous, anxious or on edge” and “not being able to stop or control worrying”. The frequency of occurrence in the past two weeks was rated from 0 = “not at all”, 1 = “several days”, 2 = “over half the days”, and 3 = “nearly every day”. Answers of the first two items were added to a total score (0 to 6); a score ≥ 3 has a good sensitivity (87%) and specificity (78%) for major depression. Cronbach alpha in the present study was = .83. A sum score ≥ 3 (range 0–6) of the other two items indicates generalized anxiety with good sensitivity (86%) and specificity (83%), performing well as a screening tool for all anxiety disorders [21]. The scale is reliable (Cronbach’s alpha in the current study = .77.) and was validated in a general population sample [20].The reliable and valid Perceived Stress Scale [22] has been used world-wide, and has been translated in 25 languages. It measured the degree to which life in the past month had been experienced as unpredictable, uncontrollable and overwhelming on a scale from 0 = “never” to 4 = “very often”. Following recommendations by the review of Lee [23] we used the 10 item scale. The German version of the scale showed good construct validity [24]. In the present study scores on the Perceived Stress Scale demonstrated acceptable internal consistency (Cronbach alpha = .84).

The Copenhagen Personal Burnout Inventory (CBI; [25]) is part of the Copenhagen Psychosocial Questionnaire assessing physical and mental exhaustion, independently from work. It assessed the frequency of six items („How often do you feel …“): “tired, physically, emotionally exhausted, unable to go on, weak and prone to illness.” The items were rated on a 5-point scale 1 = “never/ almost never”, 2 = “rarely”, 3 = “occasionally”, 4 = “often” to 5 = “always” (COPSOQ; [26]). The scale was reliable (Cronbach alpha in the present study = .91 and showed good criteria validity in a former study [27]).

Life satisfaction was operationalized by the Questionnaire on Life Satisfaction (FLZM) and social integration was measured by the brief Loneliness Scale (LS-S). The Questionnaire on Life Satisfaction FLZ^M^ [28] is a multi-dimensional self-report measure of individual life satisfaction covering eight relevant areas of life (friends, leisure time activities/hobbies, general health, income, work/ career, housing/living conditions, family life and partnership/sexuality). The sum score of all dimensions was used as an index of global life satisfaction. Respondents rated the present satisfaction with these dimensions on a scale from 1 = “dissatisfied” to 5 = “very satisfied”. The scale is valid and has been applied in diverse studies [29]. As the scale assessed conceptually different domains, the life satisfaction sum-scores indicated only sufficient internal consistency (Cronbach alpha = .70).

The brief Loneliness Scale (LS-S; [30] reliably assessed emotional and social loneliness. The three items (“how often do you feel that… “you are missing the company of others”, “being left out” and “being socially isolated”) were rated on a 5-point scale from 0 = “never”, 1 = “rarely”, 2 = “sometimes”, 3 = “often” to 4 = “very often”. Cronbach alpha in the present study was = .84.

### Statistical Analysis

For all analyses the GPS-K was used as a continuous measure. Age was categorized by decades. The age group from 14 to 29 years was grouped together, so that the different age groups had comparable sample sizes. Further, participants are expected to undergo and complete their academic and vocational training during this age period. Thus, we wanted to determine the effect of educational training, respectively student status on procrastination by comparing students and pupils with those without any ongoing educational training within the age group from 14 to 29 years. A one-way ANOVA with post hoc comparisons (Scheffé test) was conducted for group comparisons using procrastination as dependent and age groups, respectively employment status as independent variables. One-sided *t*-tests, were used to analyze group differences in regard to socio-demographic variables. Post hoc ANCOVA with age as a covariate was calculated. Associations of procrastination with distress, stress and life satisfaction and loneliness were determined by Pearson correlations. Correlations in the order of .10 were considered small, those of .30 of moderate and beyond .50 of strong magnitude [31]. A linear regression was performed defining the sum score of the GPS-K as outcome variable. Due to the exploratory nature of the analysis, the predictors were selected by a stepwise method. As this was an exploratory study alpha adjustment was not performed. Because of the large number of tests applied in this study, *p*-values should be interpreted with caution and in connection with effect estimates. We performed calculations by SPSS Version 21.0.

## Results

### Procrastination according to sociodemographic characteristics

Table 2 shows the comparisons of mean scores in the GPS-K in different sociodemographic groups. There were no overall differences in sex or education (not presented). Participants living in a partnership scored significantly lower on the GPS-K than singles. Students and unemployed participants reported higher levels of procrastination compared to employed and retired individuals. Participants with a high self-assessed tendency to procrastinate reported significantly lower incomes. As age correlated with income, age was considered as a covariate in the analysis with a significant result (*F*[1, 2442] = 19.4, *p* <.001; age *F*[1, 2442] = 100.1, *p* <.001).

**Table 2:** Procrastination in the general population according to demographic characteristics.

	*N*	*M*	*SD*	*Sig*.
**Sex**^1)^				*t*(2516) = .96; p = .34
male	1172	2.15	.66	
female	1346	2.13	.64	
**Partnership**^2)^				*t*(2472) = 7.23; p≤.001
yes	1435	2.06	.60	
no	1039	2.25	.68	
**Employment**^3)^				*F*(3.2384 = 15.35. p≤.001
Employed	1356	2.11	.62	
Student/training	172	2.50	.69	
Unemployed	149	2.45	.74	
Retired	708	2.03	.61	
**Income**^4)^				*t*(2445) = 2.59; p≤.01
<2000 €/month	1133	2.17	.66	
≥2000 €/month	1314	2.11	.63	

***Note*:** Missing data:

^1)^ *n* = 9

^2)^ *n* = 53

^3)^ *n* = 142

^4)^ *n* = 80

### Procrastination across the life span

**Fig 1:**
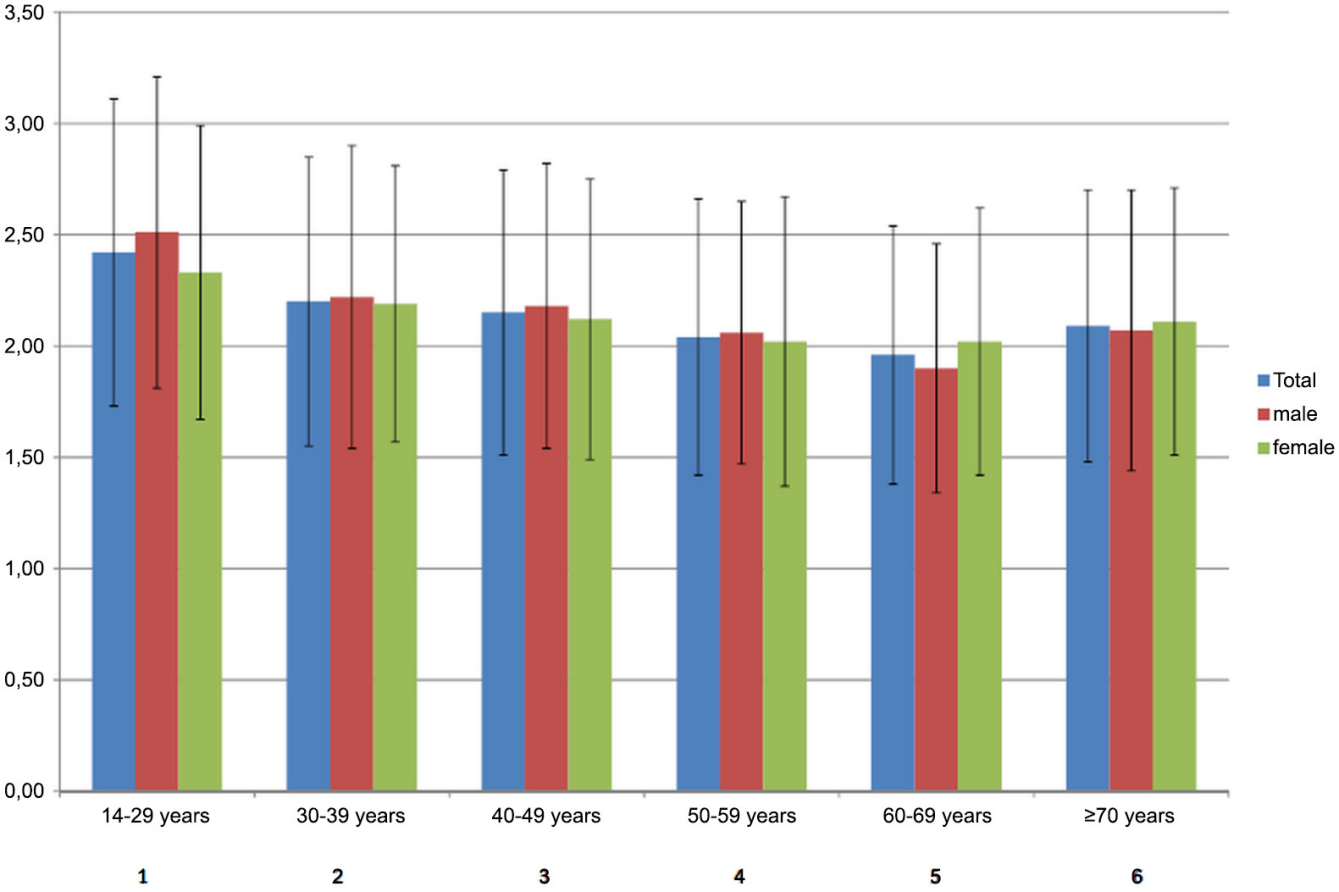
Procrastination scores across the life span according to sex. *Note*: Post hoc analyses were performed using the Scheffe’-Tests revealing 1>2–6; 2>4,5; 3>5. Age group 14–29 yrs.: *N*total = 423; *N*male = 208; *N*female = 215; 30–39 yrs.: *N*total = 339; *N*male = 153; *N*female = 186; 40–49 yrs.: *N*total = 462; *N*male = 213; *N*female = 249; 50–59 yrs.: *N*total = 501; *N*male = 229; *N*female = 272; 60–69 yrs.: *N*total = 420; *N*male = 211; *N*female = 209; ≥ 70 yrs.: *N*total = 371; *N*male = 157; *N*female = 214.

Fig 1 presents procrastination scores across the entire life span, separately for men and for women. As hypothesized, there was a significant main effect for age (*F*(5, 2504) = 27.0; *p* <.001). Post hoc analysis revealed that procrastination was highest in the youngest group (14 to 29 years) declining across the older age groups. A significant sex difference was only found in the youngest, but not in the other age groups (*t* = 2.5, *p* <.05, *d* = 0.25). In the age group from 14 to 29 years, students and pupils reported more procrastination compared to their employed peers (*t* = 2.1, *p* <.05, *d* = 0.29).

### Associations of procrastination with distress, perceived stress and life satisfaction

As Table 3 shows, all correlations were in the direction hypothesized and of low to moderate magnitude. Procrastination was most strongly associated with perceived stress followed by depression anxiety and fatigue. The association with overall life satisfaction was negative. All individual domains of life satisfaction were significantly negatively associated with procrastination (range from *r* = .13 to *r* = .31); the correlations between procrastination and satisfaction with work, respectively income were highest.

**Table 3:** Correlations between procrastination, age, income, depression, anxiety, fatigue, stress and the domains of life satisfaction (*N* = 2506–2521).

	GPS	PHQ	GAD	CBI	PSS	LS-S	FLZ
GPS	-						
PHQ	.36*	-					
GAD	.32*	.75*	-				
CBI	.27*	.60*	.59*	-			
PSS	.39*	.56*	.54*	.48*	-		
LS-S	.27*	.56*	.55*	.46*	.48*	-	
FLZ	-.35*	-.50*	-.44*	-.44*	-.48*	-.54*	-

*Note*: Results are significant at *p <.001; GPS = General Procrastination Scale; PHQ = Patient Health Questionnaire depression module; GAD = Generalized Anxiety Disorder Scale; CBI = Copenhagen Personal Burnout Inventory; PSS = Perceived Stress Scale; LS-S = Loneliness Scale; FLZ = Questionnaire on Life Satisfaction

Moderate associations were found between depression, anxiety and fatigue and between perceived stress and depression and anxiety. Moderate to strong and consistent negative correlations were found between life satisfaction, depression, and anxiety.

### Predictors of procrastination

As table 4 shows, lower age, male sex, lack of a partnership, unemployment, the presence of depression, stress and fatigue were predictive of procrastination in a multivariate analysis, explaining 24% of variance.

**Table 4:** Predictors of procrastination.

	*β*	*t*	*p*
**Age**	-.19	-10.56	.000
**Sex**	-.05	-2.53	.012
**Partnership**	.07	3.97	.000
**Unemployment**	.05	2.61	.009
**Depression**	.19	7.66	.000
**Perceived Stress Scale Short**	.24	10.46	.000
**Fatigue**	.07	2.97	.003

*Note*: *Adjusted R*^*2*^ .*24; F(7*, *2339) = 105*.*66; p = *.*0000*. Not significant in the stepwise linear regression analysis: part of Germany (East/West), education, household income, religion, work, anxiety

## Discussion

Unlike most previous studies on procrastination, we conducted a representative community survey covering a broad spectrum of mental health characteristics and life domains across the entire life span from 14 to 94 years. As hypothesized and consistent with previous studies, procrastination was highest in the youngest cohort (14–29 years). We have not found a consistent sex effect, however, only in the youngest (and most strongly procrastinating group) from 14–29 years, men procrastinated more than women. Previously inconsistent findings on sex effects may have been due to different age compositions in different samples. Procrastinating was higher among singles, in unemployed (vs employed and on pension), and students.

Cleary, procrastination, as we assessed it, was associated with a high level of perceived stress, depressiveness, anxiety, fatigue and reduced life satisfaction across a whole range of domains (work, leisure time etc.). In a multiple regression model, in addition to age and sex, lack of a partnership was a predictor of procrastination, along with unemployment, depression, perceived stress and fatigue.

How can the negative association of procrastination and age be explained? Several reasons can be hypothesized regarding the development of personality, time perception, coping styles over the life span, and cohort effects:

As reported by McCrae et al. [32], conscientiousness has increased with advancing age, a personality trait which is strongly negatively associated with procrastination [16]. According to the maturity principle [33] conscientiousness is essential for successfully achieving development tasks like assuming responsibilities of adult work and family life. Indeed, looking more closely at the youngest age group, participants undergoing education or vocational training procrastinated more than their working peers. This is an important finding as many studies on procrastination were based solely on student samples. As the high procrastination among unemployed may indicate, work schedules may provide a structure of time and demand counteracting procrastination, whereas less structured educational programs may require more active scheduling by the student or trainee leaving more opportunities for procrastination. A further explanation for our finding might be that older adults use more effective and content specific problem solving strategies than young adults [34] leading to less procrastination compared to less appropriate strategies e.g. an avoidant problem solving style [35]. Moreover, the perception of time might chance across the life span influencing the association between procrastination and age: According to Socioemotional Selectivity Theory [36] older adults perceive time as limited in contrast to younger adults, who may perceive more options, choices and chances available in their future. Interestingly, in a recent study 85 students were given a restricted time window for studying for a forthcoming exam. The restriction of available working time increased the learning efficiency and significantly reduced the tendency to procrastinate [37]. If time is perceived as more valuable and scare, individuals may use the available time by implementing instead of postponing actions. There also has been evidence that non-procrastinators tended to perceive their use of time to be more purposive and they showed higher levels in time control [1].

There may also be cohort effects, e,g. the youngest German cohort might be influenced by affluence, individual workings conditions and internet availability having changed over time. Compared to the older ones, the younger cohorts have grown up in a climate of economic and educational affluence and stability offering an increasing range of vocational and life style chances. While choice is considered as pivotal for autonomy and psychological well-being in Western countries, choice overload can induce negative outcomes like paralysis and poor decision-making [38; 39]. Likewise, in our psychotherapeutic practice we have been observing that the variety of options can be challenging for young people suffering from mental illnesses who tend to procrastinate (c.f. [40] for trait procrastinators seeking counselling). This clinical observation is in accord with previous evidence suggesting that individuals with a less developed ego identity status (e.g. diffusion status) showed higher tendencies to procrastinate in emerging adulthood, probably to avoid making fateful and identity-shaping decisions [41; 42]. Moreover, in recent years, the internet has become an integral part of everyday life and is widely used in working and educational environments. Online activities providing constant distractions from scheduled tasks may encourage procrastination and can even considered as a key aspect of problematic internet use, a phenomenon which affects mainly the young generation [43; 44].

Consistent with previous studies, we found pervasive negative associations of procrastination with successful mastery in virtually all life domains. Deliberately postponing actions can create a high level of discomfort and distress (e.g. [45]), and individuals with the tendency to procrastinate were consistently stressed, distressed and fatigued. These associations underscore the convergent and discriminant validity of the short scale of the GPS used.

Yet, in a cross-sectional study, causality cannot be determined. I.e. procrastination and associated failure may reduce self-efficacy and lead to negative consequences regarding mental health. On the other hand, depression, anxiety and fatigue may induce procrastination. Indecisiveness is a core criterion of depression. Avoidance of tackling demands out of a fear of failure is one of the hallmarks of the major anxiety disorders, which may finally compromise vocational achievement. On the other hand, prolonged unemployment may lead to reduced mental health and self-efficacy promoting procrastination.

Data were limited to self-report. Therefore the findings of the study can be considered valid for self-assessed procrastination, however, not necessarily to observable procrastinating behavior [6]. We currently have no clearly defined cut-off score of the General Procrastination Scale. While our sample was representative of the German community, we cannot preclude that people with an extreme level of procrastination may have not participated due to a lack of intrinsic motivation for action [46]. Overall, our data supported the conceptualization of procrastination as a maladaptive self-regulatory strategy. The connection between distress and procrastination in this study has important implications for identifying risk groups, who may also delay necessary medical, particularly mental health treatment [9; 12]. In further analysis we will focus on the association between procrastination and media use in adolescents and young adults which might be a major distraction, particularly for those who do not have a regular work schedule like students and unemployed. As discussed earlier, future studies exploring the possible mediating role of identity status in different age groups (not only in emerging adulthood) are required to develop a deeper understanding of the relation between procrastination and age. Given the broad negative associations of procrastination with vocational success and interpersonal integration, stress and distress, prospective studies are needed to determine developmental trajectories, identifying risk factors and mechanism of procrastination.

## Supporting Information

S1 Study Data (SAV)
